# Markerless motion tracking and correction for PET, MRI, and simultaneous PET/MRI

**DOI:** 10.1371/journal.pone.0215524

**Published:** 2019-04-19

**Authors:** Jakob M. Slipsager, Andreas H. Ellegaard, Stefan L. Glimberg, Rasmus R. Paulsen, M. Dylan Tisdall, Paul Wighton, André van der Kouwe, Lisbeth Marner, Otto M. Henriksen, Ian Law, Oline V. Olesen

**Affiliations:** 1 Department of Applied Mathematics and Computer Science, Technical University of Denmark, Lyngby, Denmark; 2 Department of Clinical Physiology, Nuclear Medicine and PET, Rigshospitalet, University of Copenhagen, Copenhagen, Denmark; 3 TracInnovations, Ballerup, Denmark; 4 Radiology, Perelman School of Medicine, University of Pennsylvania, Philadelphia, Pennsylvania, United States of America; 5 Athinoula. A. Matinos Center for Biomedical Imaging, Department of Radiology, Massachusetts General Hospital, Boston, Massachusetts, United States of America; North Shore Long Island Jewish Health System, UNITED STATES

## Abstract

**Objective:**

We demonstrate and evaluate the first markerless motion tracker compatible with PET, MRI, and simultaneous PET/MRI systems for motion correction (MC) of brain imaging.

**Methods:**

PET and MRI compatibility is achieved by careful positioning of in-bore vision extenders and by placing all electronic components out-of-bore. The motion tracker is demonstrated in a clinical setup during a pediatric PET/MRI study including 94 pediatric patient scans. PET MC is presented for two of these scans using a customized version of the Multiple Acquisition Frame method. Prospective MC of MRI acquisition of two healthy subjects is demonstrated using a motion-aware MRI sequence. Real-time motion estimates are accompanied with a tracking validity parameter to improve tracking reliability.

**Results:**

For both modalities, MC shows that motion induced artifacts are noticeably reduced and that motion estimates are sufficiently accurate to capture motion ranging from small respiratory motion to large intentional motion. In the PET/MRI study, a time-activity curve analysis shows image improvements for a patient performing head movements corresponding to a tumor motion of ±5-10 mm with a 19% maximal difference in standardized uptake value before and after MC.

**Conclusion:**

The first markerless motion tracker is successfully demonstrated for prospective MC in MRI and MC in PET with good tracking validity.

**Significance:**

As simultaneous PET/MRI systems have become available for clinical use, an increasing demand for accurate motion tracking and MC in PET/MRI scans has emerged. The presented markerless motion tracker facilitate this demand.

## Introduction

Magnetic resonance imaging (MRI) and positron emission tomography (PET) are of great importance in the diagnosis and treatment of many neurological diseases. These modalities offer unique tissue contrasts at the expense of long image acquisition duration, making patient head motion a critical problem. The degradation of image quality resulting from patient motion can potentially lead to reduced detection of clinically relevant features, negatively influencing diagnosis and treatment. It is estimated that patient motion increases the cost of MRI examinatios by $115,000 per scanner per year [1]. The problem is particularly acute in pediatric scans, where sedation and anesthesia are often used, which can lead to adverse reactions [2, 3]. To minimize the negative outcome of such head motion, various methods for motion correction (MC) has been proposed for MRI and PET reconstruction.

For MRI, prospective MC, where the imaging field of view (FOV) coordinate system is continuously updated during acquisition, has been demonstrated using a variety of tracking techniques [4, 5]. Retrospective MRI MC uses motion information retrospectively to adjust the reconstruction to compensate for motion-induced errors [5]. Unlike prospective MC, retrospective correction enables reconstruction both with and without motion corrected images.

PET only allows retrospective MC, as the acquisition cannot be dynamically adapted to compensate for motion. However, the MC can take place at different phases of the PET reconstruction, from MC of raw listmode data [6–8] to MC of the reconstructed image frames [9–11]. These MC methods are generally based on the assumption of knowing the precise head pose (position and orientation) during the scanning.

Motion information can be acquired using different sources, both directly from the acquired imaging data or using an add-on motion-tracking systems. Each approach has its own trade-off in terms of accuracy, complexity of implementation, and demands for additional hardware. Estimating motion from the imaging device itself requires no additional hardware, but can impose additional complexity on the acquisition and reconstruction of the data and may have limited time and spatial resolution at the same time. In the context of MRI, motion data are often acquired by redundant sampling patterns, either built into the imaging acquisition, or interleaved as “motion navigators” [4, 5]. In contrast, a variety of methods have been suggested for tracking markers attached to the subject. For MRI, markers have included field probes [12, 13], active markers [14], gradient sensors [15], and optical targets [16–18]. In general, markers must be attached rigidly to the subject, and different attachment strategies have been presented for each of these markers to address this challenge. Applying a stamp to the patient’s head has also been investigated as a mean to avoid the risk of marker detachment [19]. However, feature extraction from stamps or facial characteristics alone may be computationally expensive or unstable and has been demonstrated only for retrospective correction. Data-driven motion detection in PET shows promising results [11, 20]. However, it may be difficult to distinguish motion-induced changes from functional changes in tracer distribution over time. These methods resemble a limited time resolution of the motion estimation. Optical marker tracking is somewhat simpler in PET, as the line of sight to the subject is not obscured by receive coils, as in MRI, allowing more flexible marker design [21]. A markerless motion tracking approach in PET has also been demonstrated on small awake animals using head landmarks and structured light to estimate motion [22, 23]. However, the same accuracy obtained by similar marker based approaches was not achieved and the method was not applied to humans. Finally, simultaneous PET/MRI systems can also use the motion information intrinsic in the MRI data to estimate motion for both systems [24].

Until now, no external motion tracking device has been designed to be compatible with both PET and MRI scanners. Existing solutions for MRI typically require attachment to the receive coils and do not consider the location of the PET detectors. Conversely, motion trackers for PET scanners are not designed to be compatible with the strong magnetic forces acting in the MRI environment.

In this work we present and evaluate the first markerless motion tracker, Tracoline 2.0 (TCL2), addressing rigid head motion for PET, MRI, and simultaneous PET/MRI. While the first generation of Tracoline was designed for MC of PET scans only [25], a mechanical and optical redesign now makes it compatible with both PET and MRI. PET compatibility was originally achieved by positioning the hardware outside the range of the PET detectors to maintain good PET sensitivity. MRI compatibility however, requires a strict non-interfering in-bore design, such that the scanner’s strong magnetic field is not affected by any magnetic or electric components. Furthermore, all system components need to resist the strong magnetic field during operation. Finally, for MRI, the limited view of the patient’s head through the MRI coils needs to be considered.

The new hardware design comply with these conditions and the current software performance allows for accurate and real-time motion tracking. In combination, these improvements can effectively address the challenge of patient motion in both PET and MRI. Fast and reliable motion estimation are the key features for enabling real-time MC and for being a valuable tool in a clinical setting. We present results demonstrating how real-time motion tracking is applied for prospective MC in MRI and for retrospective MC of PET data reconstruction.

The motion tracker is installed and evaluated on a hybrid PET/MRI scanner during a clinical study consisting of 94 pediatric patients with brain tumors and a controlled MC study with two healthy volunteers. Volunteers are used to demonstrate prospective MC in MRI, because customized scan sequences supporting motion input are required in MRI, but are currently not available for clinical use.

The motion tracker is based on a computer vision technology using a structured light surface scanner, continuously scanning the face of the patient using a synchronized light modulator and camera. This approach requires no attachment of optical markers, reducing the clinical preparation time compared to maker-based solutions. In addition, no patient interaction is required and therefore it does not compromise patient comfort. Further, it eliminates tracking failure due to slipping markers. The system is capable of motion tracking of real patients and a tracking validity parameter (TVP) is used to ensure that the tracking is reliable and that incorrect tracking is not used for motion correction. Using incorrect tracking for motion correction may degrade the images in contrast to correcting the images, which is unacceptable for clinical use especially for prospective MC, where the images without correction does not exist. A TVP is computed for each motion estimate to accept or reject estimates in real time to ensure tracking robustness.

## Materials and methods

### The motion tracker

TCL2 is the first markerless motion tracker, addressing the challenge of rigid head motion during PET and MRI neuroimaging. It is designed to be compatible with both PET and MRI modalities and to provide reliable real-time motion feedback during scanning. In order to comply with the restrictive strong magnetic environment inside the MRI scanner room, TCL2 had to be significantly redesigned compared to the first version of the Tracoline motion tracker [7, 25]. MRI compatibility is achieved by replacing all in-bore electronics and objectives with non-magnetic and non-electric components. The motion tracker requires a good view of the patient’s head in order to make a 3D facial surface reconstruction. Therefore, to maintain a good in-bore FOV, 3 meter long optical image fibers are introduced to extend the view from the camera and light source, both placed out-bore behind the scanner and away from the personal and patient. The optical fibers thereby act as flexible vision extenders, creating a remote 3D surface scanner. The fragile fibers are enclosed in an energy chain cable carrier to protect them from mechanical injuries. At the end of the optical fibers in-bore, a vision probe facing the patient contains the image objectives, configured to have a focus distance between 10-25 cm. All the out-bore electronics are enclosed in a radio-frequency (RF)-shielded box. The box contains the potentially RF-emitting and ferromagnetic camera and light source. The box is custom made with a 9 mm wooden frame with the inside surfaces of the box are covered with 1 mm RF-shielding copper and all optical fibers pass though waveguides to avoid electromagnetic radiation. 5/12 V DC power is supplied from the adjacent control room through a standard Siemens RF wall filter and data are transferred via an optical cable through a waveguide to the control computer located in the adjacent room.

PET compatibility requires the in-bore vision extenders not to interfere with the in-bore PET detectors encircling the patient’s head. The in-bore vision probe is therefore located slightly behind the PET detection area and tilted to an angle allowing for a clear view of the patient through one of the head coil openings.

**Fig 1:**
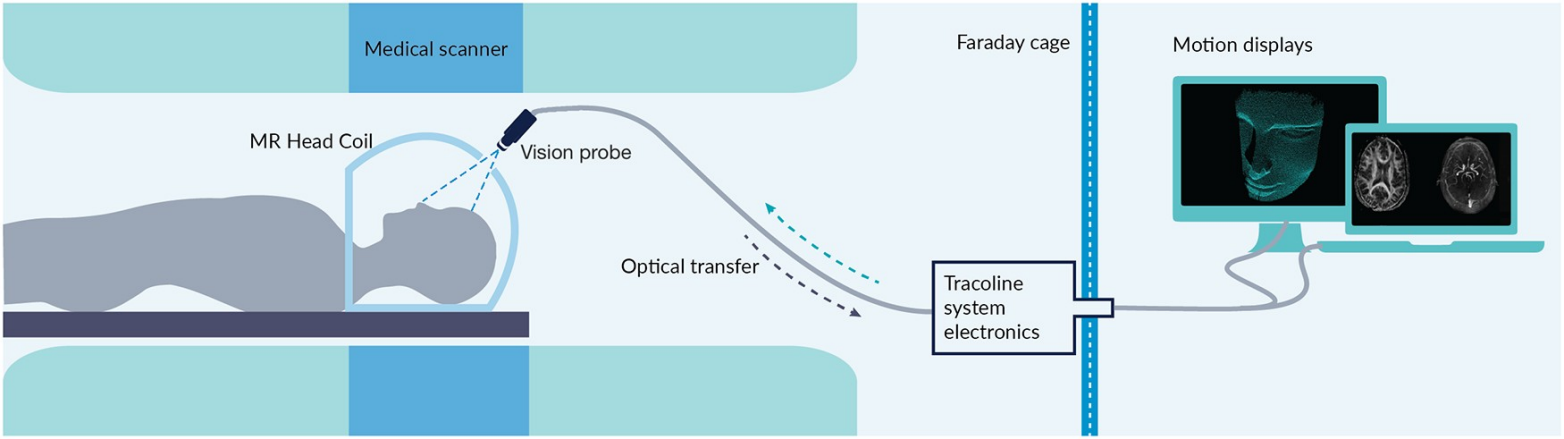
Tracoline 2.0 system integrated with the scanner. Sketch of the developed Tracoline 2.0 system integrated with the mMR Biograph hybrid 3T PET/MRI.

The integration of the system with a PET/MRI scanner is conceptually illustrated in Fig 1. The motion tracker is installed with an mMR Biograph hybrid 3T PET/MRI scanner (Siemens Healthineers, Erlangen).

**Fig 2:**
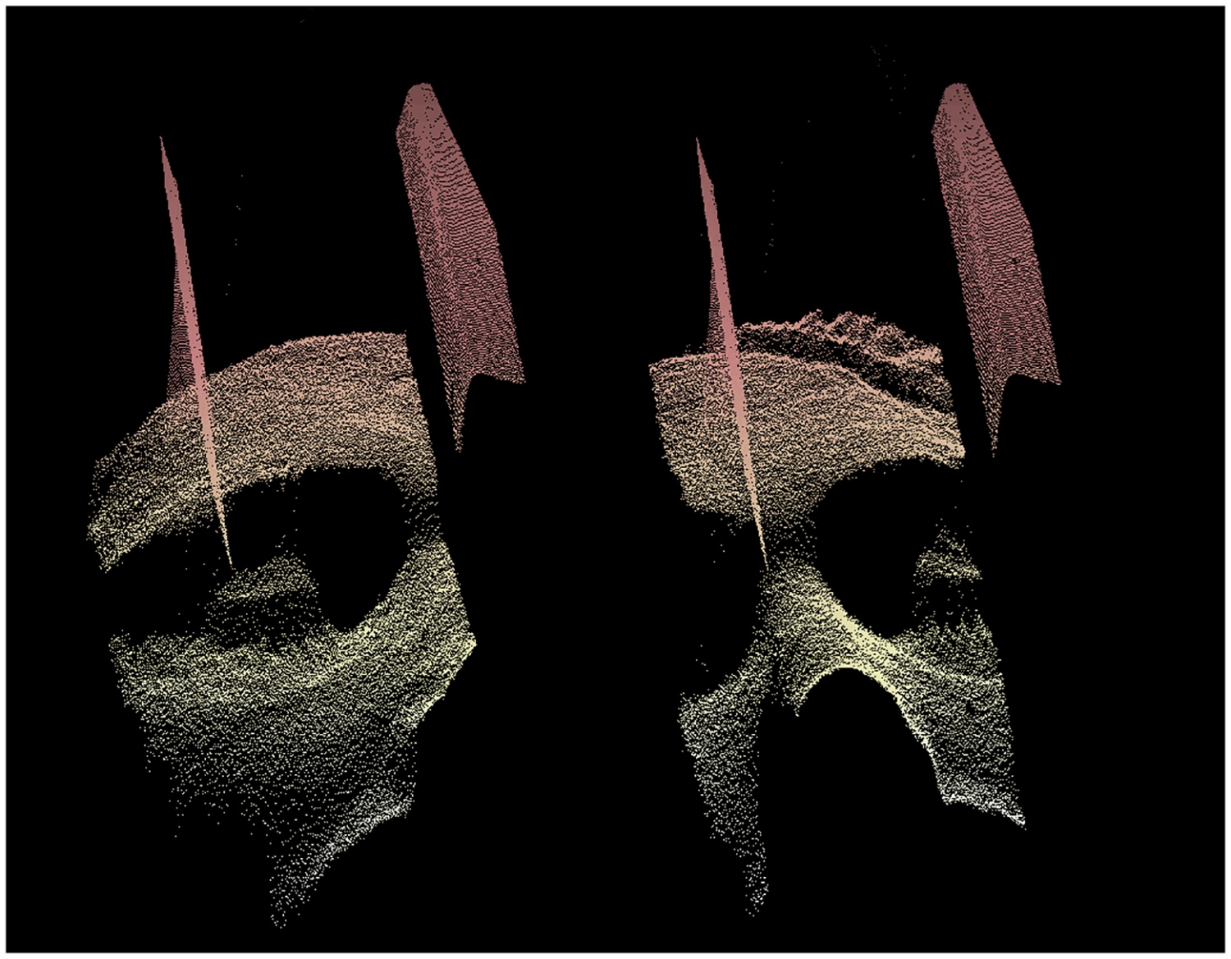
3D facial point clouds of a subject. 3D facial point clouds of a subject inside an MRI head coil obtained during MRI acquisition using the TCL2 for motion tracking. The two point clouds are obtained at different times during the acquisition. Part of the MRI head coil is seen in front of the facial surface of the patient. It is clearly visible, that the head of the subject has moved relative to the head coil during the acquisition.

TCL2 uses a synchronized camera and light source to continuously scan the surface of the patient’s head. The surface scanner produces high-resolution 3D point clouds consisting of thousands of individual points. Non-visible infra-red structured light is used to avoid patient discomfort [26]. The continuous stream of surface scans creates 30 point clouds per second. Each of these point clouds are used for geometric alignment relative to an initial reference position during the scanner image acquisition. Examples of a 3D point cloud can be seen in Fig 2. In this figure, parts of the head coil surface is visible in front of the patient’s face. This stationary head coil within the FOV is treated as an inactive foreground segment and hence not included in the motion tracking procedure.

The TCL2 motion tracking software package (TracSuite) has also been updated and improved in terms of performance and reliability, to comply with the real-time tracking constraint of prospective MC. The motion tracking software was re-implemented for fast and parallel shared-memory multiprocessing. TracSuite estimates the patient’s head pose using an Iterative Closest Point (ICP) algorithm [27]. The head pose is computed at a frequency of 30 Hz, a sample rate which is sufficient for most MC purposes.

The ICP alignment algorithm imposes the assumption of tracking a rigid surface. Occlusion, rapid motion or facial movements may violate such a rigidity assumption. Also, if the patient moves outside the FOV, accurate tracking is not possible. Therefore, the estimated pose is analyzed against the reference point cloud, in order to determine how reliable the tracking result is. A TVP ∈[0, 1], is introduced to indicate the tracking validity of each head pose. When the TVP value is equal or close to 1, it indicates high tracking validity. If the TVP becomes 0, the pose estimate is rejected and the previous pose will be used as a more reliable pose instead.

### Data

To demonstrate the compatibility of the motion tracker in a clinical PET and MRI environment, TCL2 was used for motion tracking in pediatric patients with presumed brain tumors. A total of 94 pediatric patient scans (median age 10.1 years, range 0.1-19.5 years) included in a clinical PET/MRI study using ^18^F-FET were all obtained between March 2015 and January 2018 using an mMR Biograph hybrid 3T PET/MRI scanner (Siemens Healthineers, Erlangen). The pediatric scans consist of one 40 minutes PET acquisition and six MRI scans, where two of the MRI sequences are acquired after injection of intravenous contrast medium Gadovist. Sequence type and parameters of the six MRI sequences are given in Table 1. The study was approved by The Regional Research Ethics Committees of Capital Region of Denmark (ID: H-6-2014-095) and registered at clinicaltrials.gov (NCT03402425). All patients or their parents gave written informed consent for participation in the study and a subset of the patients has been included in previous pulications [28, 29].

**Table 1:** List of MRI sequences used in the pediatric scans.

Sequence	Parameters	Voxel Size [mm]
T1 MPRAGE	9° flip angle; TR/TE/TI 1900/2.52/900 ms	1.00x1.00x1.00
T1 TIRM	150° flip angle; TR/TE/TI 2000/34/800 ms	0.45x0.45x4.00
T2 FLAIR	130° flip angle; TR/TE/TI 9000/95/2500 ms	0.43x0.43x4.00
DWI	180° flip angle; TR/TE 5600/61 ms	1.15x1.15x4.00
T2 Blade(GD)	140° flip angle; TR/TE 4000/118 ms	0.72x0.72x5.00
T1 MPRAGE(GD)	9° flip angle; TR/TE/TI 1900/2.52/900 ms	1.00x1.00x1.00

(GD), Contrast enhanced sequences. Contrast medium Gadovist.

#### PET data

To demonstrate the use of TCL2 for correcting PET scans for motion artifacts, two of the pediatric PET patients were chosen, patient (a) and (b), for which the tracked motion data indicated rather large motion fluctuations and minor motion fluctuations, respectively. Patient (a) was selected to highlight the effect of MC for large motion while patient (b) was included to confirm that there is no reduction in image quality after MC for patients exhibiting small motion. The patients were scanned using ^18^F-FET tracer, advantageous in PET imaging for brain tumor suspected patients, with a PET acquisition duration of 40 min. The scans were reconstructed using a dynamic framing, with matrix size 172 × 172 × 127, a Gaussian filter with full width half maximum: 3.5 mm, 3.5 mm, and 5 mm, and with respective frame durations of 6 × 10 s, 4 × 15 s, 2 × 30 s, 2 × 60 s, 2 × 150 s, and 4 × 300 s, resulting in a total of 22 frames for each patient according to our standard clinical procedure. Further for the scans, a static frame from 20-40 min was reconstructed with matrix size 344 × 344 × 127 and a Gaussian filter size of 3.5 mm, 3.5 mm, and 5 mm.

#### Prospective MRI data

Customized scan sequences supporting motion input were required for prospective MC of MRI. These were not currently available for clinical use. Moreover, prospective reconstruction of MRI disables the possibility of image reconstruction with and without MC for comparison. Therefore, to demonstrate the ability to support prospective MC in MRI, two healthy 24 year old male volunteers were studied in a separate method development study. They were both scanned using the same hybrid PET/MRI scanner as the pediatric patient group, to again demonstrate compatibility of both modalities. Each subject was scanned four times. In two of the scans, subjects were instructed to remain motionless (referred to as a *No motion* case), while in the other two scans they were instructed to perform a repeatable motion pattern during the scan (referred to as a *Motion* case). These four scans were divided into two with- and two without-MC-enabled conditions (referred to as *MC ON* and *MC OFF* cases, respectively). In the motion routine, subject (a) was instructed to do moderate drift-like movements, whereas subject (b) performed large motions to demonstrate the measuring volume of the tracking system. The subjects were scanned using a custom 3D FLASH sequence that can apply prospective MC based on the motion estimates from TracSuite [15], and the motion was tracked using TCL2. The 3D FLASH sequence has the following parameters: FOV 256 mm × 256 mm × 56 mm with 1 mm × 1 mm × 2 mm voxels (phase/read/slice directions respectively), bandwidth of 1000 Hz/pixel, TR of 15 ms, TE of 2.68 ms, and flip angle 35 degrees. A Siemens mMR Head/Neck 16-channel coil was used and no parallel acceleration was applied.

### PET motion correction

The PET MC was conducted retrospectively using a customized version of the Multiple Acquisition Frame method incorporating subframes according to the tracked motion. The MC pipeline was implemented around the e7-reconstruction-tools from Siemens. A frame of PET data were chosen from the pool of listmode data. This frame was initially divided into several subframes according to a given threshold of motion (THM), where the individual subframe locations are equal to the patient pose at the given time of acquisition. The attenuation map (*μ*-map) was repositioned to align the individual subframes. The specified subframing and the aligned *μ*-map were given as inputs to the e7-tools performing the reconstruction using the ordered subset expectation maximization (OSEM) algorithm [30]. The reconstructed subframes from e7-tools were afterwards rotated and translated according to the motion data to a common pose of a chosen reference frame. Finally, the subframes were merged into a single motion corrected and reconstructed frame, using a duration-weighted average. In case of a dynamic scan, this method was conducted across all the dynamic frames.

The procedure of defining a subframe was constrained to comply with two conditions: A minimum of true counts must be available within the subframe to avoid lack of statistical information, and a THM must be achieved. A lower limit of true counts was found to be 3 million corresponding to a sufficient amount of applicable statistical information and a 2 mm THM was chosen, based on our experience.

#### Evaluation of PET motion correction

The dynamic PET reconstructions were evaluated with respect to PET tracer distribution over time (i.e. across a sequence of PET frames), where a time-activity curve (TAC) was drawn for the mean radioactivity in a given high uptake region of interest (ROI) with a size of approximately 0.2 cm^3^. The tracer uptake in tissue is expressed as standardized uptake value (SUV) by dividing the radioactivity (kBq/mL) in the tissue by the radioactivity injected per gram of body weight [31]. The resulting TACs consists of 22 data points representing mean SUV values for each of the 22 frames in the dynamic reconstruction. This evaluation was conducted for TACs representing reconstruction with and without MC enabled.

For the TACs it was expected to see an increase in SUV, i.e. an increase in image intensity in the given ROI, for the motion corrected images, due to correction for the motion induced artifacts reducing image intensity in high uptake regions. Also a decrease in motion related noise in the TACs reprecenting MC was expected [32].

### Prospective MRI motion correction

The TCL2 control unit was connected to the internal MRI scanner Ethernet network. The system control unit sets up an ethernet server, which accepts pose requests from the scanner and replies with the current head pose. The server automatically converts the head poses to the scanner device coordinate system (DCS), before sending them to the scanner. A 3D FLASH sequence was modified to request the current patient head pose from the TCL2 control once per TR. The sequence performs prospective MC every TR by adjusting the gradients, RF pulse frequencies and k-space sample phase every TR, in relation to the changes in the patient’s head pose [15].

### Cross calibration

For external tracking systems a cross calibration is required, describing the transformation between the tracking coordinate system (TCS) and the scanner DCS. TCL2 returns for each time interval a rigid transformation representing the overall relative motion of the tracked surface. Here, we use the Euler representation for rotations and utilize that a transformation is a simple multiplication of rotation and translation in homogeneous coordinates. The transformation matrices are of the form

$$\begin{array}{c} \text{A}=[\begin{array}{cc} \text{R} & \text{d}\\ 0 & 1 \end{array}], \end{array}$$ (1)

where **R** denotes the 3 × 3 matrix of rotations and **d** is the 3 × 1 vector of displacements. The cross calibration is described through multiplication of two transformations

$$\begin{array}{c} {\text{A}}_{\text{DCS}2\text{TCS}}={\text{A}}_{\text{DCS}2\text{PCS}}{\text{A}}_{\text{PCS}2\text{TCS}}\;, \end{array}$$ (2)

where PCS denotes the coordinate system relative to the patient. The transformation **A**_DCS2PCS_ is defined according to the positioning of the patient in the scanner. In device coordinates of the PET/MRI system, using the head-first-supine patient orientation, this transformation is

$$\begin{array}{c} {\text{A}}_{\text{PCS}2\text{DCS}}=[\begin{array}{cccc} 1 & 0 & 0 & 0\\ 0 & -1 & 0 & 0\\ 0 & 0 & -1 & 0\\ 0 & 0 & 0 & 1 \end{array}]. \end{array}$$ (3)

The system software provides a semi-automatic cross calibration tool, where a point cloud of the tracked facial surface is first manually repositioned to roughly align to a point cloud of the patient’s head extracted from an MRI scan. In these studies a clinical MPRAGE sequence was used with the following parameters: FOV 512 mm × 512 mm × 192 mm with 1 mm × 1 mm × 1 mm voxels (phase/read/slice directions respectively), bandwidth of 179 Hz/pixel, TR of 1900 ms, TE of 2.4 ms, flip angle 9 degrees and an aquisition duration of 5:02 min. Afterwards, the ICP alignment procedure finds a transformation from TCS to PCS, **A**_PCS2TCS_. Prior to scanning, the vision probe can be moved to adjust the FOV to the patient’s face. With a mobile system such as TCL2, a cross calibration is required each time the position of the vision probe changes.

For MC application of a tracking device in PET/MRI brain imaging, the rigid transformations describing conversion of tracking in TCL2 coordinates to tracking in scanner device coordinates at time *t*, is expressed as

$$\begin{array}{c} {\text{A}}_{\text{DCS}}\left(t\right)={\text{A}}_{\text{DCS}2\text{TCS}}{\text{A}}_{\text{TCS}}\left(t\right){\text{A}}_{\text{DCS}2\text{TCS}}^{-1}\;, \end{array}$$ (4)

where **A**_TCS_(*t*) describes the transformation of the point cloud in TCL2 coordinates.

## Results

### PET and MRI compatibility

The motion tracker has been installed and in use with the combined PET/MRI scanner during normal clinical routines for a period of more than two and a half years. In this period, the motion tracker were operated by unsupervised radiographers for the majority of all the examinations. During this period no problems related to compatibility between the scanner and the motion tracker were reported.

Motion tracking data were recorded during the clinical study for all 94 pediatric patients. Out of these 94 examinations, 34 patients received anesthesia during the scan. The average root mean square (RMS) motion of the 34 examinations was 2.00 mm, compared to an average RMS motion of 5.27 mm for the 60 patients not receiving anesthesia. A t-test shows that the anesthetized patients have significant lower RMS motion with a p-value < 0.001.

The majority of the motion data has been computed with high tracking confidence, as 88 of the scans contain no rejected tracking estimates. The remaining six scans contain one or more rejected tracking estimates due to a low TVP. For five of these cases, the rejected tracking estimates occurred only during rapid motion with a duration for each rejection period never exceeding one second. The number of rejections corresponds to approximately 0.1% of the total number of estimates for these five cases. The final case contains a large proportion of rejected estimates of almost 15%. This loss is primarily caused by substantial motion and a non-optimal reference point cloud containing little information for the alignment procedure.

### PET motion correction

**Fig 3:**
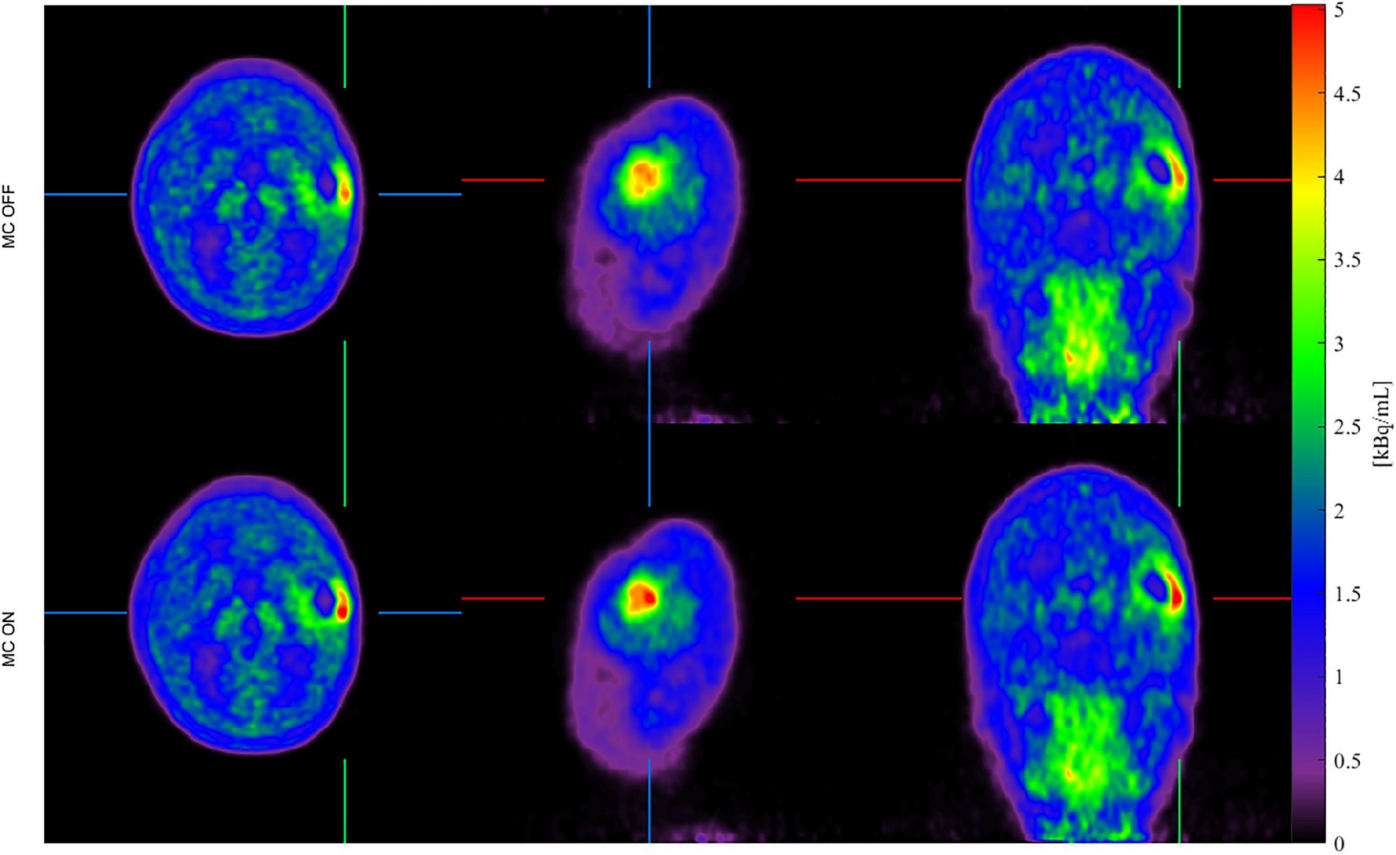
MC of 20-40 min. static PET image of patient (a). Static ^18^F-FET PET image (20-40 min. after tracer injection) of patient (a). Top-to-bottom rows show axial, sagittal and coronal image slices for image reconstructions without motion correction (left column) and with motion correction (right column). It is seen that the intensity of the tumor has increased after MC is applied.

**Fig 4:**
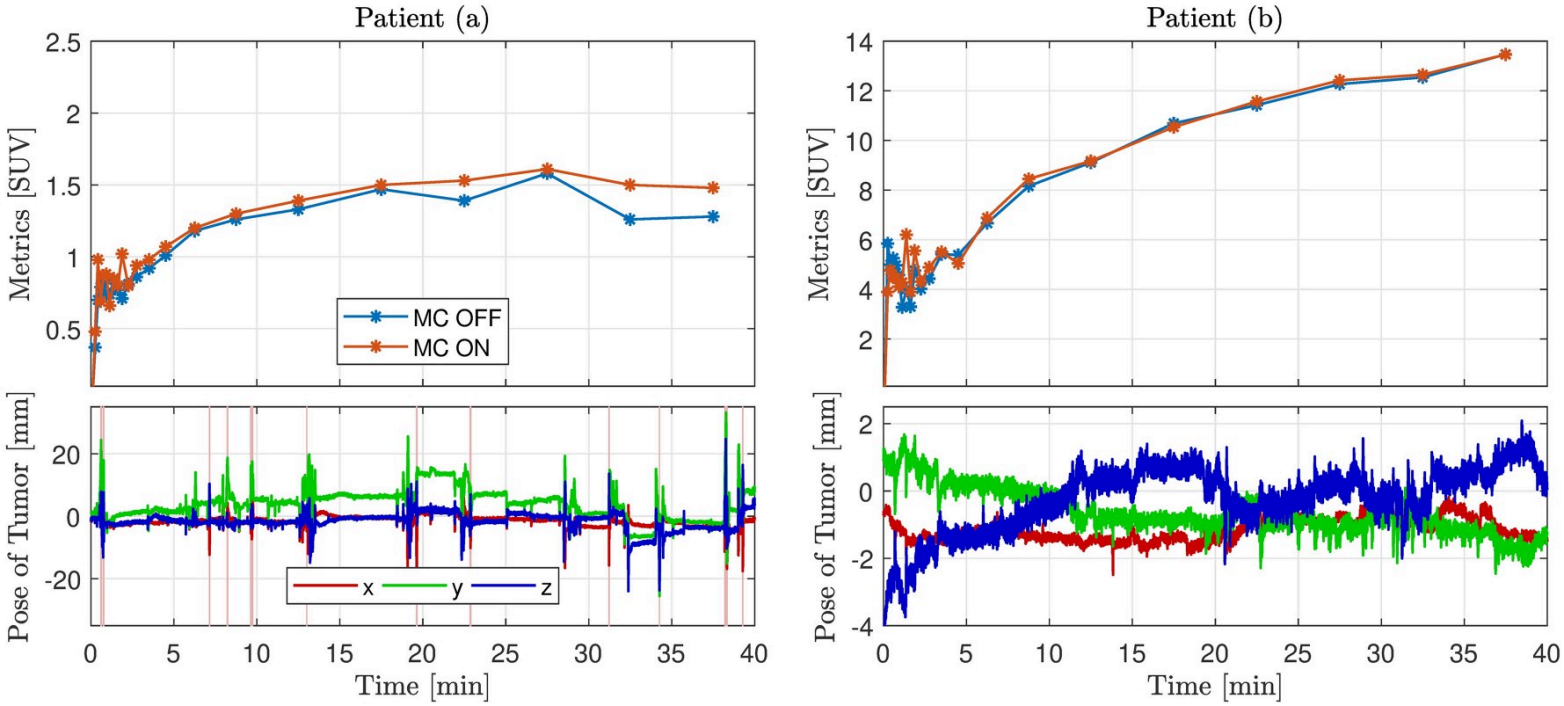
Time-activity curves and tumor motion of patient (a) and (b). Time-activity curves (TACs) and motion tracking curves corresponding to PET patients (a) and (b). For each patient the top plot indicates the TACs representing the mean standardized uptake value in a given tumor region of interest over time (i.e. for the respective dynamic frames). Reconstruction curves with MC and without MC are shown. Each point in a TAC corresponds to the beginning of a frame. The bottom plot shows the motion tracking curves indicating position of the tumor point along the x-, y- and z-axis. 149 tracking estimates are rejected due to low tracking validity for patient (a), marked by the vertical red lines. There are no rejected tracking estimates for patient (b). It is noted that the small rapid fluctuations of the motion curves are caused by respiratory motion as shown in Fig 5.

Fig 3 shows image slices of a static (single frame) 20-40 min reconstruction of the 40 min dynamic ^18^F-FET PET scan of patient (a) with and without MC. The reconstruction with MC is conducted using a subframing with a 2 mm THM. The scan shows increased uptake of ^18^F-FET in a brain tumor in the left hemisphere. Within the scan range from 20 to 40 min, the patient exhibits head movements giving rise to the tumor motion seen from the motion tracking curves for patient (a) in Fig 4. The resulting effect of the MC is an increased intensity in the ROI in already high intensity regions compared to the images reconstructed without MC. This effect matches the expectation, i.e. that patient motion during a PET brain scan will result in images with blurred high intensity regions constituting a larger area with lower intensity, compared to images obtained from scans with no patient motion (cf. [32]). It is noted that in the coronal image slice, another tendency is seen in the upper neck region, however this is not a part of the brain and can not be considered rigid. During the 40 min scan 147 tracking estimates were rejected due to low tracking validity. These rejections occur only when the patient moves rapidly and the average total tumor 3D displacement before each rejected interval is 17 mm. None of the rejected estimates are used for the motion compensated image reconstruction.

**Fig 5:**
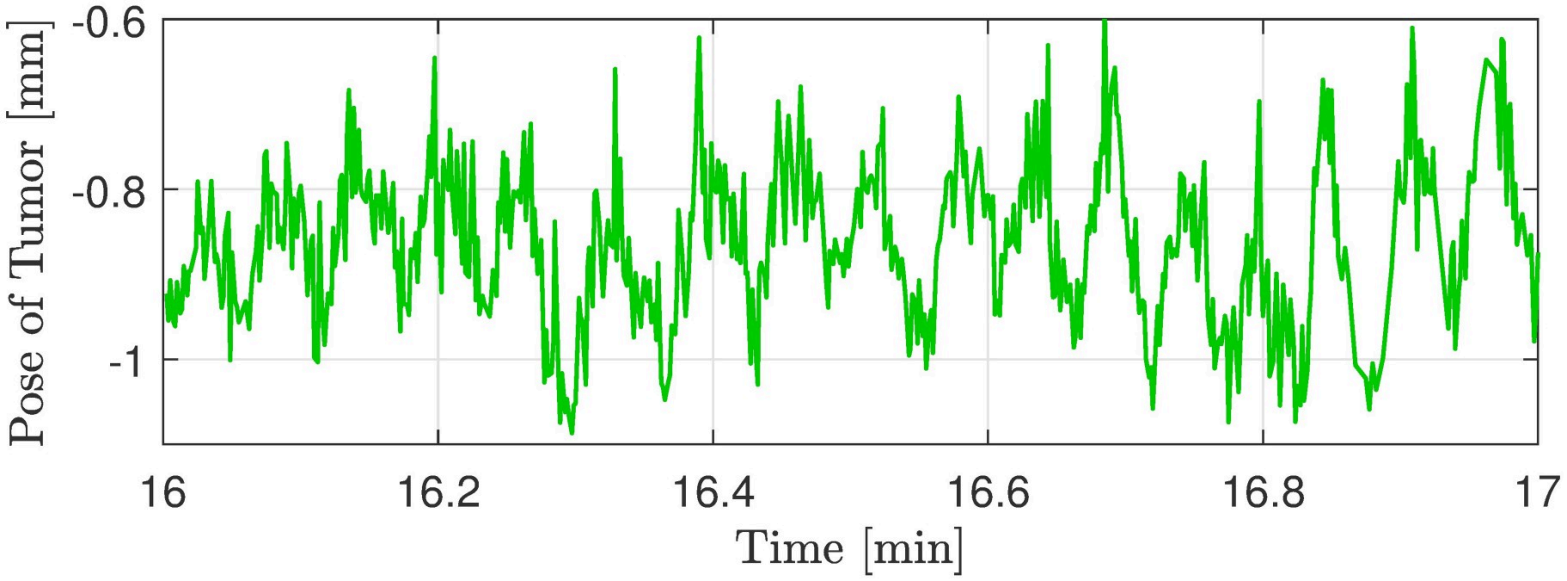
Magnification of tracking curve. Magnification of the y-direction tracking curve from patient (b) seen in Fig 4. The periodic oscillations are the respiratory motion performed by the patient.

The TACs for the dynamic reconstruction of the pediatric PET patients are shown in Fig 4 together with the motion of the examined tumor. The TAC represents a tracer increase and washout over time, resulting in a curve rising from the time of injection, and afterwards continuing to rise or decrease depending on the relationship between washout and uptake in the tumor. This curve pattern is seen from all the TACs in Fig 4. However, the motion corrected image for patient (a) yields a more smooth curve, which indicate that motion related noise has been reduced. For patient (b) the differences between MC and no MC are minor in accordance with the small detected motion. The considerable fluctuations of all TACs within the first three minutes can be explained by the low signal-to-noise ratio caused by low frame duration and low tracer uptake resulting in few true counts.

The TAC investigations indicate, that for patient (a), the MC reconstructed image has higher SUV metrics compared to the non-motion corrected image. The frames corresponding to the largest effect of the MC also correspond to time intervals with large motion fluctuations. This substantiates the effect of the MC.

### Prospective MRI motion correction

**Fig 6:**
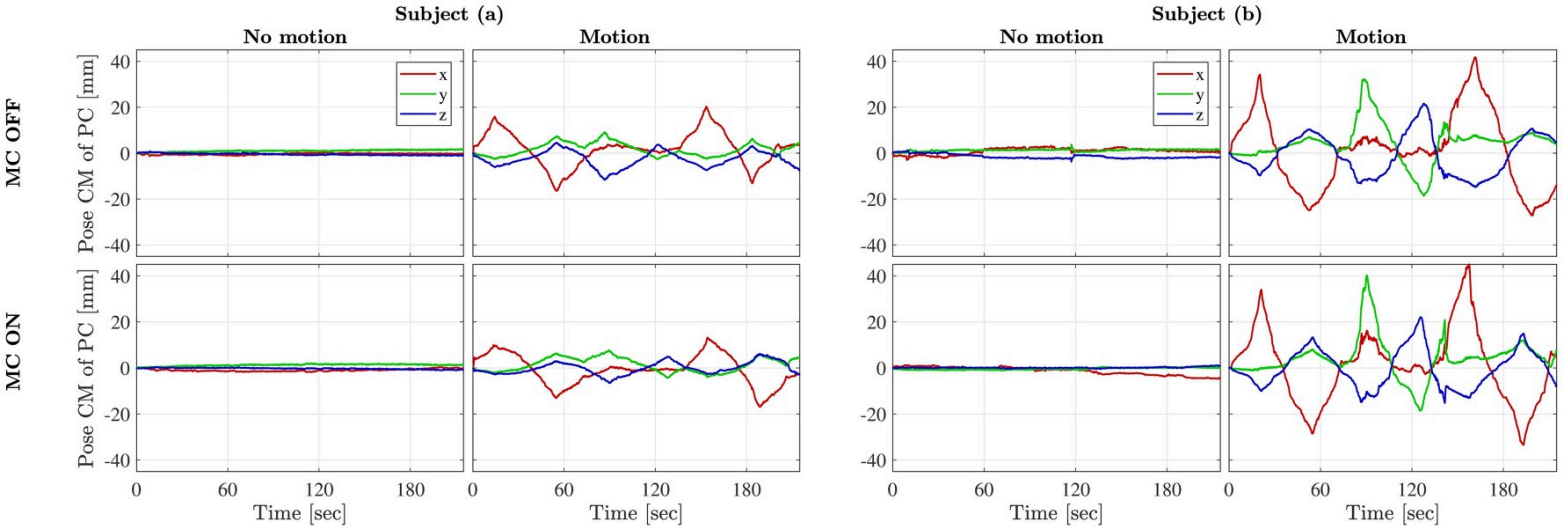
Motion of subject (a) and (b) from the four studied cases. Plots of the position of the center of mass (CM) of the 3D point cloud (PC) representing the motion of subject (a) and (b). The top row corresponds to the scans without motion correction and the bottom row to the scans with motion correction enabled. The plots show that the subjects were able to replicate the motion pattern between the scans with and without motion correction.

**Fig 7:**
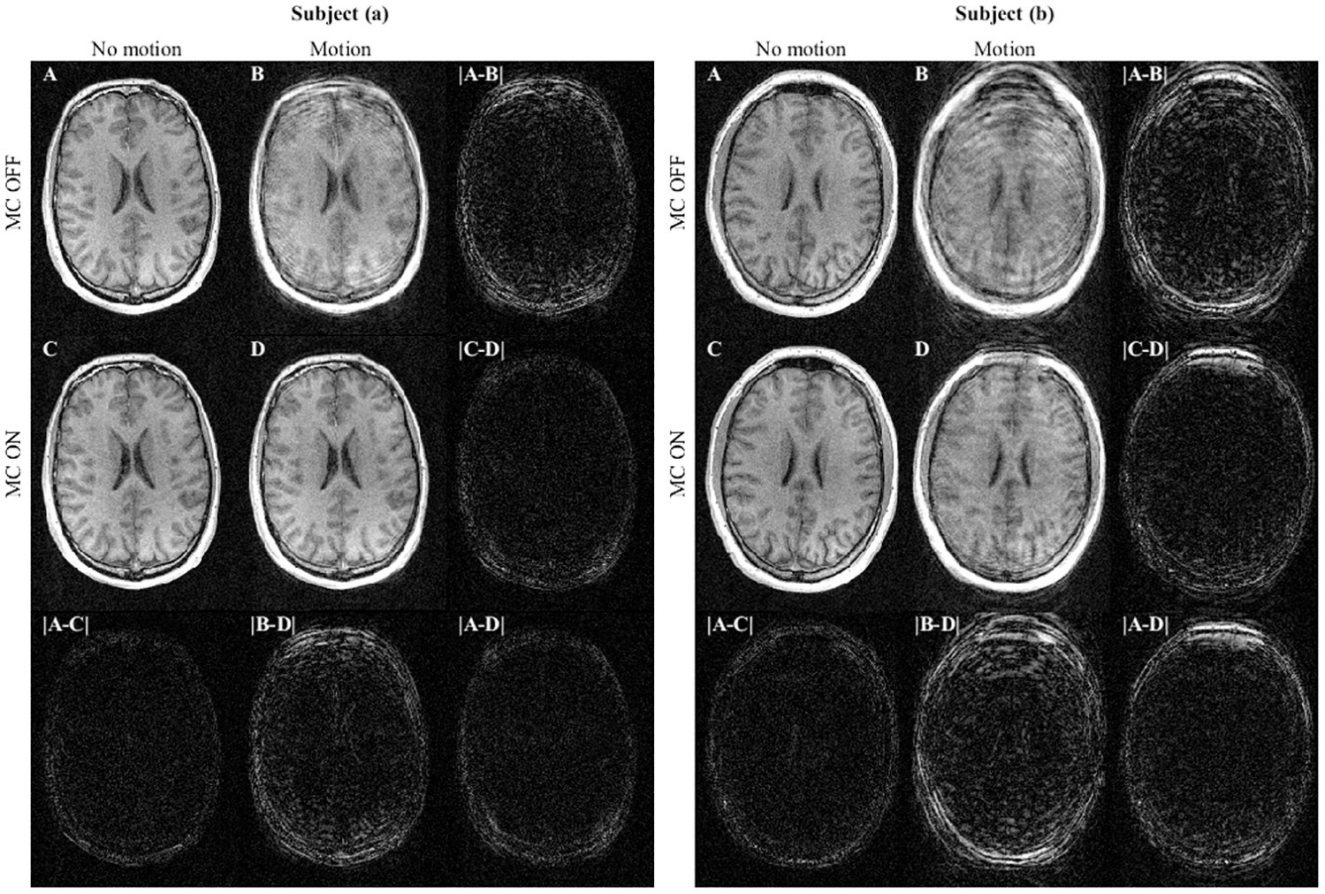
Axial image slices from the 3D FLASH scans of subjects (a) and (b). The motion performed during the acquisition corresponds to the graphs in Fig 6 subject (a) and (b), respectively. The four scan images representing scans with and without motion and with and without motion correction (MC): **A**: *No motion, MC OFF*, **B**: *Motion, MC OFF*, **C**: *No motion, MC ON*, and **D**: *Motion, MC ON*. The absolute difference between scans are present in the last row and column for both subjects. These are visualized using the same mapping from pixel intensities to grey scale values as in the MRI images. For both subjects image **B** show degraded image quality resulting from motion. Image **D** shows the improved image quality as a result of the prospective MC.

Fig 6 shows the performed motion corresponding to the MRI images of subject (a) and (b) shown in Fig 7. It is noted that the subjects have repeated the motion pattern between the scans with and without MC. Fig 7 shows the axial image slices of the 3D FLASH scan obtained from subject (a) and (b) respectively in the four cases: **A** *No motion, MC OFF*, **B** *Motion, MC OFF*, **C** *No motion, MC ON*, and **D** *Motion, MC ON*. Image **B**, **C** and **D** are all registered to image **A**. In the same figure, absolute difference images are visualized.

Motion artifacts are clearly visible from image **B** for both subjects and are evidently reduced after MC (cf. image **D**). For subject (a), the motion corrected image is similar to the non-motion corrupted image, however some artifacts remain after MC for subject (b) as expected for such large motion. This is reflected in the |**A-D**| images too.

Images for subject (a) in Fig 7 indicate that the TCL2 system is capable of substantially improving image quality with motions up to ±10-15 mm. When subject motions approached ±40 mm, images for subject (b) indicate that only incomplete correction was achieved.

Importantly, we see that the MC does not degrade the quality of scans without motion. This is consistent with what is seen from the difference image |**A-C**| for both of the subjects.

When it comes to *No motion, MC ON*, the differences are negligible, as it is the case for *Motion, MC ON* for subject (a). As shown, deviations are visible in the skull for subject (b) in the *Motion, MC ON* case (cf. difference image |**A-D**|, but reduced in comparison to the *Motion, MC OFF* case (cf. difference image |**A-B**|) for the same subject.

## Discussion

We have demonstrated the first markerless motion tracker compatible with simultaneous PET and MRI. The system has been robust for tracking motion in real patients while acquiring both MRI and PET. Only few cases, where the patients moved rapidly, lead to situations where correct tracking poses could not be estimated. These tracking poses were properly detected by the TVP algorithm. A visual inspection of the point clouds and the rejected transformations for patient(a), confirms that some of these tracking estimates would not represent a correct transformation. In addition, none of the accepted tracking estimates are notably misaligned. In just one of the 94 pediatric scans, a sub-optimal reference point cloud was created leading to additional tracking rejections. A subsequent re-alignment with an updated point cloud improved the TVP, such that 4% of the tracking estimates were rejected instead of 15%. For all other patient scans, only five cases contained rejected tracking estimates, with rejection periods of less than one second each. In total, an acceptance rate of more than 99.9% of all the motion tracking estimates is achieved, indicating the tracking robustness of the motion tracker. The clinical feasibility of the motion tracker is evidenced by the robustness of tracking when the system is operated by radiographers in the normal clinical settings.

Results also show that the motion tracker is feasible for retrospective MC of PET and prospective MC of MRI with notable image improvements. The MC PET study of the two pediatric ^18^F-FET PET patients with brain tumors demonstrated an enhancement of image contrast after MC in case of ±10 mm motion. In PET imaging no ground truth exists of how the functional images should actually appear, as it is the case in MRI imaging resembling anatomy. This makes it more difficult to quantify the impact of MC, as it was considered unethical to conduct two scans in the included patients, i.e. with and without motion. Nevertheless the time-activity curve and its progress can give a quantitative estimate of the MC. A TAC investigation showed that for large motions, a difference in SUV of up to 19% between the corrected and non-corrected TACs was detected for certain frames. As an example, further investigation of the patient (a) seen in Figs 3 and 4 led to an increase in SUV values in the ROI after MC, which would change the interpretation of the scan and thereby have an actual impact in the clinical course for the patient in question. In the case of tumor motion in the ±2-3 mm range, e.g. as seen for the patient (b) in Fig 4, the MC pipeline dose not introduce errors related to the MC.

In the MRI study, the motion corrected images were noticeably improved compared to the non-corrected ones. TCL2 was able to track extreme motions, where subject (b) was asked to perform the largest possible motion inside the head coil with a slit width of 6 cm. For subject (b) there were remaining artifacts despite correction for motion. These artifacts are likely in part the result of B_0_-field inhomogeneities induced by the extreme head motion, as B_0_ field shimming is conducted before each scan assuming no motion within the scan. Additional artifacts may also have been induced by the motion of the subject relative to the B_0_ receive fields of the coil elements, which is not accounted for in the prospective motion correction sequence we used. The study also shows, that the impact of MC in the case of no motion is minor, as seen from the difference images |**A-C**| in Fig 7. The minor differences are likely caused by scan-to-scan variations and small motions despite the subjects’ attempt not to move (cf. *No motion* plots of Fig 6).

In the present work we have demonstrated the application of our MC system to 3D FLASH data. This sequence was chosen because of it is a key component of many high-resolution structural imaging protocols, providing both good T1-weighted contrast and low distortion. However, 3D sequences, particularly those whose total scan time extends to minutes, are known to be acutely sensitive to subject motion. As such, this sequence makes a useful exemplar of the efficacy of the TCL2 motion tracker. While the present demonstration is not exhaustive of all the possible ways an optical tracking system such as ours could be used in MRI, other groups have previously employed optical trackers (usually with markers and/or not compatible with PET) for prospective correction. Those results have demonstrated that the direct, prospective correction of the FOV and excitation pulses can reduce motion-induced artifacts in 3D scans using parallel acceleration [33] as well as 2D EPI- and TSE-based sequences [4, 17, 34, 35].

**Fig 8:**
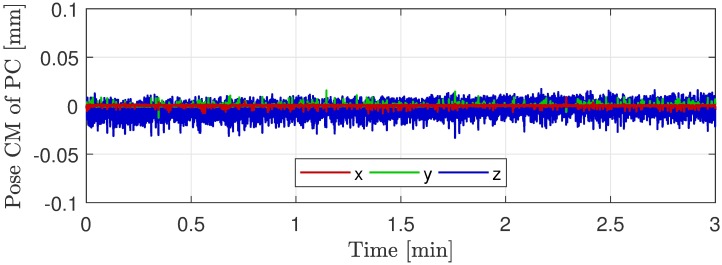
Tracking of stationary face phantom. Tracking curves represented by center of mass of the 3D point cloud (PC) obtained from a TCL2 tracking of a stationary face phantom during an mMR Biograph MRI scan.

TCL2 demonstrated sufficient sensitivity to detect respiratory motions of size ±0.5 mm, as seen from Fig 5, showing a magnification of the tracking curve for patient (b). This indicates a tracking sensitivity substantially better than the general MRI image resolution. In this relation, investigations of the TCL2 tracking were made. A stationary face phantom was tracked in the mMR Biograph during a similar scan situation to the clinical scans. The tracking curves seen in Fig 8 shows the low tracking noise level of the system. The tracking has maximal fluctuations of ±0.026 mm and a standard deviation of 0.0089 mm. These investigations substantiate the high sensitivity of TCL2 and indicate, that the respiratory fluctuations are in the order of 16 times the noise level. It is noted, that fluctuations of the tracking curve of the z-direction is significantly larger compared to the two other directions. Immediately this is explained by relatively parallel images axes of the vision system with the depth measure almost aligned with the scanner z-direction.

The cross calibration, aligning the tracking and the scanner coordinate systems, is easily conducted with a standard sequence. This makes the system mobile and simple to set up with different kinds of scanners. The FOV of the vision probe is adjusted to fit each patient regarding the wide span of patient size and figure (as an example, some of the pediatric patients do not reach as far into the headcoil and lies further down compared to the adult patients). This procedure takes approximately 10s and is the only additional manual adjustment for each patients. The TCL2 requirement of a cross calibration makes the system dependent on a non motion corrupted MRI sequence. This dependence could be minimized by introducing a fast acquirable MRI sequence only containing what is essential to the cross calibration. The MPRAGE sequence used in these studies aquire the total head volume, however only image data of the patients’s facial surface is necessary as an input to the cross calibration. The scantime can hence be reduced through reduction of the FOV of the MRI sequence.

In this work an MRI compatible motion tracker has been demonstrated to robustly estimate rigid head motion during MRI, PET, or PET/MRI examinations. The system is designed for head motion tracking, and the motion tracker is therefore only evaluated for brain imaging. However, the tracking system may be able to track motion of other body parts provided that the motion is relative rigid and that the body part has sufficient surface features to allow a unique rigid transformation between a current PC and the reference PC for accurate motion estimation in all directions.

## Conclusion

We have presented the TCL2 motion tracker, performing real-time markerless motion tracking and monitoring compatible with both PET and MRI scanners. Design optimization makes the motion tracker feasible for the MRI environment while not compromising PET sensitivity. It is the first time that a motion tracking system has been successfully demonstrated in simultaneous PET/MRI for brain imaging including prospective MC of MRI. For both modalities, a reduction in motion-induced artifacts has been achieved after MC. The system demonstrates robust motion tracking using a TVP to avoid invalid tracking from being used in critical situation such as prospective MC. During the clinical study of 94 pediatric patients, 88 scans had no rejected tracking estimates, while five scans had only minor dropouts. The tracking capabilities of motions ranging from small respiratory motions to the largest possible motion within the MRI head coil have been demonstrated.
